# New Interventional Therapies beyond Stenting to Treat ST-Segment Elevation Acute Myocardial Infarction

**DOI:** 10.3390/jcdd8090100

**Published:** 2021-08-24

**Authors:** Pablo Vidal-Calés, Pedro L. Cepas-Guillén, Salvatore Brugaletta, Manel Sabaté

**Affiliations:** 1 Institut Clínic Cardiovascular, Hospital Clínic, Universitat de Barcelona, 08036 Barcelona, Spain; pvidal@clinic.cat (P.V.-C.); cepas@clinic.cat (P.L.C.-G.); sabrugal@clinic.cat (S.B.); 2 Institut d’Investigacions Biomèdiques August Pi i Sunyer (IDIBAPS), 08036 Barcelona, Spain; 3 Centro de Investigación Biomédica en Red. Enfermedades Cardiovasculares (CIBERCV) CB16/11/00411, 28029 Madrid, Spain

**Keywords:** ST-segment-elevation myocardial infarction, primary percutaneous intervention, reperfusion injury, microvascular dysfunction, infarct size, cardioprotection

## Abstract

Myocardial infarction remains the principal cause of death in Europe. In patients with ST-segment-elevation myocardial infarction (STEMI), a promptly revascularization with primary percutaneous intervention (PCI) has transformed prognosis in the last decades. However, despite increasing successful PCI procedures, mortality has remained unchanged in recent years. Also, due to an unsatisfactory reperfusion, some patients have significant myocardial damage and suffer left ventricular adverse remodeling with reduced function—all that resulting in the onset of heart failure with all its inherent clinical and socioeconomic burden. As a consequence of longer ischemic times, distal thrombotic embolization, ischemia-reperfusion injury and microvascular dysfunction, the resultant myocardial infarct size is the major prognostic determinant in STEMI patients. The improved understanding of all the pathophysiology underlying these events has derived to the development of several novel therapies aiming to reduce infarct size and to improve clinical outcomes in these patients. In this article, based on the mechanisms involved in myocardial infarction prognosis, we review the new interventional strategies beyond stenting that may solve the suboptimal results that STEMI patients still experience.

## 1. Introduction

Myocardial infarction is the leading cause of death and disability in Europe. Primary percutaneous coronary intervention (PCI) has dramatically improved prognosis of patients with ST-segment-elevation myocardial infarction (STEMI) over the last decades [1] and it is considered the cornerstone of STEMI treatment [2]. However, mortality remains high and has reached a plateau in the last years (7–8% of cardiac mortality) [3]. Also, among STEMI survivors, there is a significant group of patients who suffer a suboptimal myocardial reperfusion, developing left ventricular (LV) remodeling and subsequent heart failure (HF) in the long-term, with all the implicit morbidity and socioeconomic burden. Notably, 20% of patients are hospitalized with HF in the first year post-STEMI [4,5].

The principal factor related to long-term clinical outcomes in STEMI patients is the infarct size, which has been strongly associated with both all-cause mortality and HF hospitalization [6]. At the same time, several factors play an important role on infarct size extension: ischemic time, distal embolization, microvascular dysfunction, and reperfusion injury [7]. All these mechanisms are related to infarct size and ultimately worse outcomes so they have been studied as therapeutic targets.

To address this unmet therapeutic need, multiple pharmacological and interventional strategies have been developed in recent years. These novel techniques aim to reduce infarct size, to preserve ventricular function and to change prognosis of STEMI patients. In this review, we provide an overview of different innovative interventional therapies—beyond primary PCI—for potentially improving outcomes in STEMI patients.

## 2. Prognostic Determinants in Stemi

Among various factors that define prognosis in STEMI patients, infarct size after reperfusion is perhaps the most important. Infarct size is related to the extent of necrotic areas upon which an adverse myocardial remodeling is generated—and thus ventricular dysfunction leading to subsequent HF is the main outcome. HF is a prevalent entity after STEMI—13% of patients at 30 days and 20% at 1 year after discharge. It has an increasing clinical and socioeconomic impact due to the large number of hospitalizations [4,8]. As it has been demonstrated, infarct size is associated with HF: every 5% increase in infarct size increases the risk of hospitalization for HF by 20% [6].

The magnitude of the cardioprotective therapies efficacy has been assessed by measuring the infarct size with different means—ECG changes, biomarkers, single-photon emission computed tomography (SPECT), and more recently, cardiac magnetic resonance (CMR). The infarct size depends on the area at risk (AAR), which is the perfusion territory of the occluded artery. The only way to quantify the myocardial salvage obtained with these therapies is comparing the infarct size to the AAR. However, we have no available and generally validated in vivo measure of the AAR in the STEMI patients. In fact, the myocardial salvage assessment remains controversial, even with new proposals such as the T2-weighted CMR hyperintensity to delineate the AAR [9]. Nowadays, the mass of new late gadolinium contrast enhancement on CMR (as a percentage of LV mass) is the strongest measurement of infarct size.

Major surrogate determinants of STEMI prognosis (all involved in infarct size) are discussed below.

### 2.1. Ischemic Time

Currently, one of the fundamental dogmas in STEMI treatment is that “time is cardiac muscle”. A prompt PCI is clearly associated with a reduction in mortality risk [10]—every minute of delay in PCI affects prognosis [11]. Specifically, a door-to-balloon time > 90 min was related to a higher in-hospital mortality [12]. Consequently, the “less than 90 min” door-to-balloon time is considered the objective in the current guidelines with a level of evidence IA [2]. In this context, increased efforts to shorten door-to-balloon time have been applied to all patients with STEMI in the last years. Although ischemic time has been shortened during the years, mortality has remained unchanged. Therefore, further efforts to reduce door-to-balloon time may not improve outcomes. Probably, other factors that affect the total ischemic time could be refined in the future and potentially impact prognosis, such as awareness of symptoms, reducing transfer time between medical facilities or time of symptom onset to treatment [5].

### 2.2. Distal Embolization

Coronary distal embolization derived from the rupture or erosion of an atherosclerotic vulnerable plaque may occur in STEMI either spontaneously or iatrogenically during PCI. This phenomenon occurs frequently—11% of patients with STEMI treated with PCI—and it is also associated with increased risk of HF development [13]. The thrombotic debris arrive to the coronary microcirculation and perpetuate thrombosis by inducing platelet aggregates. Besides, there is secretion of pro-inflammatory, vasoconstrictor, and cytotoxic substances. All these processes lead to arrhythmias, contractile, and microvascular dysfunction [14].

### 2.3. Reperfusion Injury

A rapid and effective reperfusion is the main goal in STEMI treatment. However, paradoxically, flow restoration may produce myocardial damage by itself—a phenomenon called ‘myocardial reperfusion injury’. The pathophysiology underlying myocardial reperfusion injury includes many cellular events (increased mitochondrial permeability and intracellular calcium, oxidative stress, neutrophil activation) that lead to edema, hemorrhage, microvascular obstruction, and finally cardiomyocyte death [15]. Reperfusion injury is common in STEMI (30–40% of patients) and it is associated with mortality. Therefore, strategies targeting reperfusion injury have been developed. Nevertheless, due to its complex pathophysiology and the rapid manifestation in the first minutes after flow recovery, it has been, up to date, challenging to effectively improve outcomes in STEMI patients [16].

### 2.4. Microvascular Dysfunction

Microvascular obstruction is present when there is a persistent perfusion defect despite a normal coronary flow in epicardial arteries. This ‘no-reflow’ phenomenon affects coronary microcirculation (<200 μm diameter) and it is recognized in angiography in a considerable number of patients—1 out of 5 STEMI patients treated with PCI [17]. Besides ischemia produced by coronary occlusion, microvascular obstruction is intimately related to reperfusion injury and distal embolization. Several factors contribute to its etiology: microembolization, platelet activation, myocardial, and endothelial edema. Ultimately, in many cases there is a severe endothelial damage which generates intramyocardial hemorrhage due to extravasated red blood cells [18]. Because of this, microvascular obstruction facilitates adverse myocardial remodeling and ventricular dysfunction [19,20]. Moreover, based on data extracted from different randomized clinical trials, microvascular obstruction—identified by CMR in the first week after STEMI—is associated with infarct size, HF hospitalization and mortality. Every 1% increase in microvascular obstruction increases the risk of one-year all-cause mortality by 14% and one-year HF hospitalization by 8% [7]. As a result, microvascular obstruction is considered an attractive endpoint complementary to infarct size to be further evaluated in clinical trials regarding optimized treatment of STEMI patients [21].

**Figure 1:**
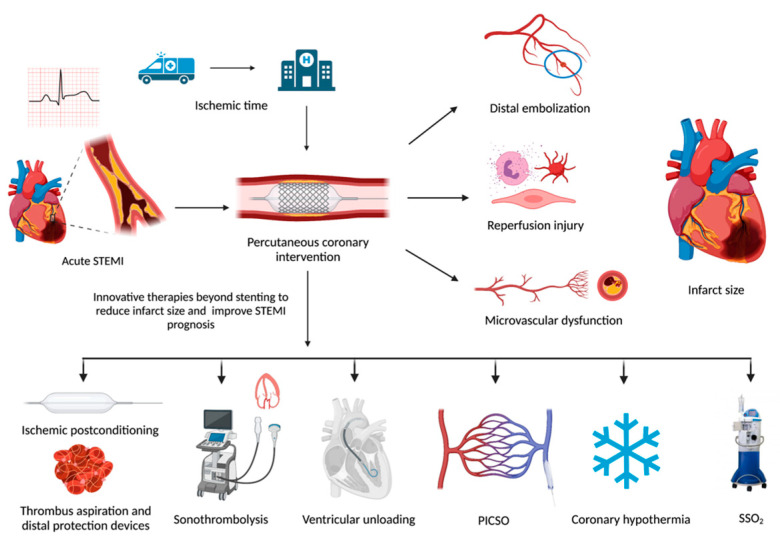
Prognostic determinants involved in infarct size extent and novel interventional strategies to enhance myocardial recovery in STEMI patients. STEMI: ST-segment-elevation myocardial infarction; PICSO: Pressure-controlled intermittent coronary sinus occlusion; SSO_2_: Supersaturated oxygen. Created with BioRender.com.

The knowledge of these pathophysiological mechanisms has led to the development of new therapies focused on reducing infarct size and improve clinical care of STEMI patients (Figure 1).

## 3. Therapies to Prevent Distal Embolization

Many pharmacological therapies (antiplatelet and antithrombotic drugs) are used in order to reduce distal embolization and optimize stenting techniques in STEMI [2]. Also, some mechanical strategies have been developed to reduce the microembolization damage by removing (aspiration thrombectomy), dissolving (sonothrombolysis) or even restricting the migration (distal protection devices) of these large thrombotic volumes.

### 3.1. Aspiration Thrombectomy

Thrombectomy aimed to reduce the extent of thrombotic burden by removing the thrombus before stent deployment. Thrombus aspiration has been widely discussed in recent years following encouraging initial results [22].

In the Thrombus Aspiration during Percutaneous coronary intervention in Acute myocardial infarction Study (TAPAS) thrombus aspiration showed better tissue reperfusion, ST-segment resolution and significant reduction in all-cause mortality (4.7% vs. 7.6%; *p* = 0.042) compared with the PCI alone group [23]. However, large multicenter randomized clinical trials have not proven any clinical benefit of this strategy in the STEMI treatment. The Thrombus Aspiration in ST-Elevation Myocardial Infarction in Scandinavia (TASTE) trial studied 7244 patients and concluded that routine thrombus aspiration before PCI compared with PCI alone did not reduce 30-day mortality (2.8% vs. 3.0%; *p* = 0.63) [24] or one-year mortality (5.3% vs. 5.6 %; *p* = 0.57). Neither re-infarction, stent thrombosis, or hospitalization for heart failure had differences between two groups [25]. The Trial of Routine Aspiration Thrombectomy with PCI versus PCI Alone in Patients with STEMI (TOTAL) study randomized 10,732 STEMI patients and confirmed lack of efficacy of manual thrombus aspiration. Furthermore, this study raised concerns about the safety of this technique; there was evidence of increased stroke within 30 days in the thrombectomy group (0.7% vs. 0.3%; *p* = 0.02) [26]. However, the association with acute ischemic stroke during PCI remains controversial [27]. Nowadays, routine aspiration thrombectomy during PCI is considered to have no benefit on clinical practice—class III recommendation [2].

### 3.2. Sonothrombolysis

Transthoracic high mechanical index (HMI) impulses from a diagnostic ultrasound transducer are used in the diagnosis of myocardial perfusion defects during a continuous microbubble infusion. These HMI impulses induce microbubble cavitation that create shear forces capable of dissolving epicardial and microvascular thrombi in STEMI animal models [28,29,30].

The first human study to demonstrate a therapeutic effect of sonothrombolysis was carried out in 30 patients that were randomized to intermittent HMI impulses prior to PCI and 30 min post-PCI (*n* = 20) or to low mechanical index (LMI) imaging only (*n* = 10) before and after PCI. In this study, intermittent diagnostic HMI transthoracic impulses (administered during an intravenous commercially available ultrasound contrast agent infusion) was safe and effective in the improvement of epicardial angiographic flow and recovery of microvascular function [31]. A larger study with 100 STEMI patients randomized in a 1:1 fashion proved that the HMI therapy before and after PCI improved angiographic recanalization (48% vs. 20%; *p* = 0.001), reduced infarct size (29 ± 22 g vs. 40 ± 20; *p* = 0.026) and increased ejection fraction after revascularization (*p* = 0.03) and at six months follow-up (*p* = 0.015) [32]. Another study, the SONOSTEMILYSIS (NCT04217304), is currently recruiting 60 high-risk STEMI patients that are expected to receive reperfusion therapy with fibrinolysis as part of a pharmacoinvasive approach with a randomization to sonothrombolysis vs. standard therapy alone.

With the emergence of this novel treatment, even portable ultrasound is going to be studied for safety and feasibility of sonothrombolysis in the ambulance between STEMI diagnosis and transfer to PCI center [33].

### 3.3. Distal Protection Devices

Distal embolic protection devices were designed to protect the microcirculation from embolization once the phenomenon occurs. These devices can be safely deployed in coronary arteries to restrict distal embolization of atherosclerotic debris at the time of PCI. A filterwire system proved clinical benefit during stenting of stenotic venous grafts [34].

However, results in the STEMI setting have been disappointing. In the Enhanced Myocardial Efficacy and Recovery by Aspiration of Liberated Debris (EMERALD) study distal protection failed to show improvement in ST-segment resolution, infarct size or the six-month composite endpoint of major adverse cardiac events (10.0% vs. 11.0%; *p* = 0.66) [35] in patients treated with distal protection system compared with the conventional PCI group. In the Drug Elution and Distal Protection in ST-Elevation Myocardial Infarction (DEDICATION) trial 626 STEMI patients were randomized and similar results were obtained: no differences in ST-segment resolution, infarct size or major adverse cardiac and cerebral events at one month (5.4% vs. 3.2%; *p* = 0.17) [36]. In conclusion, distal protection devices are not recommended for the treatment of STEMI patients.

## 4. Ischemic Postconditioning

Ischemic postconditioning—transient interruption of myocardial reperfusion by short ischemia/reperfusion cycles with an occluding balloon during early reperfusion—was described in a canine model for the first time in 2003 [37]. This cardio-protective strategy preserved endothelial function and even showed a reduction in infarct size by 44%.

In human studies, the established protocol implies four 1-min cycles of inflation/deflation with a coronary angioplasty balloon following PCI. Ischemic postconditioning proved cardiac protection (reduction in myocardial infarct size assessed by creatine kinase release) for the first time in the clinical setting with 30 patients in 2005 [38]. In another study with 50 STEMI patients, the post-conditioned group had less myocardial edema and smaller infarct size in the CMR study (13 g/m^2^ vs. 21 g/m^2^; *p* = 0.01) compared with the control group [39]. By contrast, many subsequent studies in recent years found no differences between groups in infarct size [40,41,42]. Even meta-analyses have conflicting results on the impact of post-conditioning on infarct size [43,44]. Furthermore, another meta-analysis—reviewing 1545 patients of different RCTs—revealed a null benefit of this technique on clinical outcomes [45].

Ischemic post-conditioning beneficial effect was first characterized in preclinical studies. However, this beneficial effect was not reflected in the clinical use, probably due to the high number of cofounding factors present in the clinical practice. Remarkably, cardioprotective effects with this technique are weaker in older patients [46]. Also, many co-morbidities—including hypertension, dyslipidemia, diabetes, or obesity—and some medications, such as beta-blockers, statins or antidiabetic drugs; are related to loss of cardioprotection [47]. Furthermore, some procedural aspects might add confusion to the post-conditioning results. One of the risks with performing coronary balloon inflation in the culprit lesion is the risk of embolization. The direct-stenting technique (without prior balloon dilatation of the stenosis) reduces coronary microembolization while allowing a reperfusion through residual stenosis—thus decreasing reperfusion injury. Its role as a confounder has been discussed and remains very controversial since it might maximize the differences between groups when used more frequently in the post-conditioning group [46,47].

The largest clinical trial using ischemic postconditioning (DANAMI 3-iPOST trial) tested 1234 STEMI patients and also failed to reduce all-cause death, re-infarction, and hospitalization for heart failure. However, in a post-hoc study of this population (based on the possible interference of thrombectomy on the effects of post-conditioning) there was a reduced risk of all-cause mortality and hospitalization for heart failure (10% vs. 18%; *p* = 0.016) in the patients not treated with thrombectomy [48]. This is the first trial that shows a possible cardio-protective effect of ischemic postconditioning on clinical endpoints (65% relative risk reduction of all-cause mortality). Based on these results, the iPOST2 trial (NCT03787745) will investigate the effect of postconditioning in STEMI patients without thrombectomy on the development of heart failure and death.

## 5. Left Ventricle Unloading

Left ventricle unloading prior to reperfusion may reduce the extent of myocardial necrosis by reducing oxygen consumption, improving coronary microcirculation, and decreasing reperfusion injury [49].

### 5.1. Intra-Aortic Balloon Counterpulsation

Intra-aortic balloon counter pulsation (IABC) mechanically increases coronary perfusion, reduces myocardial oxygen demand and reduces left ventricle afterload [50]. Preclinical animal studies have shown that ventricular unloading with IABC prior to reperfusion reduced infarct size compared with post-reperfusion or reperfusion alone [51,52].

Based on these results, the Counterpulsation to Reduce Infarct Size Pre-PCI for Acute Myocardial Infarction (CRISP-AMI) trial randomized patients with high-risk anterior STEMI without cariogenic shock. However, infarct size was not significantly different in the IABC plus PCI group vs. PCI-alone group (42.1% vs. 37.5%; *p* = 0.06) and no clinical benefit was found [53]. In a meta-analysis with randomized clinical trials—including 1054 patients—evaluating the benefit of IABC in patients with STEMI without cardiogenic shock; counterpulsation failed to reduce death, congestive heart failure or reinfarction. In fact, an increased risk of cerebrovascular accident was noted (2% vs. 0.3% *p* = 0.03) [54]. Currently, IABC pump is not recommended for routine use in STEMI patients.

### 5.2. Assist Devices

Several devices are able to pump flow from the left ventricle to the arterial system; including percutaneous axial-flow pumps that are easily inserted through the femoral artery and placed across the aortic valve. The most recent versions of these pumps can reach flows of 5 L/min; which may help unloading the ventricle by reducing oxygen consumption and increasing the systemic arterial flow—thus improving coronary flow [55].

These devices have mostly been used as a circulatory support in STEMI patients with hemodynamic instability due to cardiogenic shock. However, there is less evidence in patients without cardiogenic shock. In 2008, the safety and feasibility of Impella LP2.5 support (during 3 days) was tested in 20 STEMI patients without hemodynamic compromise. Left ventricle unloading with Impella in 10 patients was safe and resulted in better recovery compared with the standard group [56].

In order to promote myocardial salvage by attenuating the reperfusion injury, Kapur et al. explored in an animal model the hypothesis that first mechanically reducing left ventricle wall stress with an axial-flow catheter-based pump—while intentionally delaying coronary reperfusion injury—limits infarct size and activates cardio protective signalling pathways. This study demonstrated for the first time that initially reducing myocardial oxygen demand with Impella CP and then delaying reperfusion for 1 h reduces infarct size not just by simply decreasing oxygen demand but also by increasing cardioprotective molecular signaling [57]. This research has challenged the paradigm of STEMI treatment by suggesting that ventricle unloading prior to reperfusion may promote myocardial recovery.

Therefore, this preclinical work led to the first exploratory study in humans testing safety and feasibility of left ventricle unloading with Impella CP pump before reperfusion—a concept approach known as “delayed reperfusion”—in STEMI patients without cardiogenic shock. Obviously, this disruptive method raises some concerns not only regarding the technical safety and feasibility of pump implantation but also in the inherent delay in ischemic time. The DTU-STEMI pilot trial (Door-to-Unload in STEMI Pilot Trial) is a multicenter randomized trial where 50 anterior STEMI patients received ventricle unloading: 25 followed by immediate reperfusion and 25 with a 30-min delay to reperfusion. The technique was safe with a similar 30-day MACCE rates in both delayed and immediate reperfusion groups (12% vs. 8%; *p* = 0.99). Also, the technique was feasible in a short time interval—the start of the procedure to activation of the Impella CP required 15.4 ± 8.4 min on average and the door-to-balloon time of 84.4 ± 27.6 min. Moreover, there was no different between groups in infarct size, microvascular obstruction and ejection fraction [58].

On the basis of these conclusions, an appropriately powered pivotal trial (the STEMI-DTU pivotal trial: NCT03947619) is currently recruiting patients (up to 688 patients) to compare left ventricle unloading for 30 min with delayed reperfusion versus standard PCI with immediate reperfusion [59].

The cardioprotective effects of the LV unloading before reperfusion have been consistent with transvalvular pumps [49]. However, the role of other circulatory support therapies such as Tandem Heart or venoarterial extracorporeal membrane oxygenation (VA-ECMO)—alone or combined with Impella—remains to be defined.

## 6. Supersaturated Oxygen

Hyperbaric oxygen or supersaturated oxygen (SSO_2_) consists on the internal infusion of hypoxemic blood to the myocardium during an acute myocardial infarction immediately after coronary reperfusion. The supersaturated oxygen—with a PaO_2_ of 760 to 1000 mmHg—is delivered in the infarct-related coronary artery to promote myocardial healing. In experimental animal models, this hypoxemic blood showed a reduction in infarct size by several mechanisms: decrease in capillary endothelial cell swelling, inhibition of leucocyte activation and adherence, improvement of microvascular function and nitric oxide synthase expression, and reduction of lipid peroxide radicals [60].

Following promising first clinical experiences with intracoronary infusion of hyperbaric oxygen therapy [61], 269 patients with anterior or large inferior STEMI undergoing successful PCI within 24 h of symptoms were randomized in the AMIHOT I trial to SSO_2_ or control. Although infarct size was not significantly different between groups, in anterior STEMI patients reperfused in the first 6 h there was less infarct size and better left ventricular ejection fraction [62]. Therefore, a second randomized trial of SSO_2_ therapy (AMIHOT II) was performed focusing in these selected patients. In this case, a 90-min post-PCI infusion of SSO_2_ significantly reduced infarct size (measured by tc-99 sestamibi single-photon computed tomography) and was non-inferior in MACCE at 30 days compared to control group (5.4% vs. 3.8%; *p* = 0.77). However, two major adverse events were observed with this technique: hemorrhagic complications (related to the use of multiple arterial sheaths) and a concerning trend to stent thrombosis (4.1% of SSO_2_ patients vs. 2.5% of control patients; *p* = 0.73)—due to SSO_2_ delivery through an indwelling catheter in the stented region of the coronary artery [63].

As a result, a prospective non-randomized multicenter single-arm study (IC-HOT) tried a new ‘optimized’ SSO_2_ therapy in 100 anterior STEMI patients. The blood was withdrawn from the femoral artery and circulated via a roller pump through an extracorporeal oxygenator in a polycarbonate chamber (as originally described). Then, the catheter was placed in a different position (in the origin of the left main coronary artery) and the flow rate was higher (100 mL/min) so that the total duration is 60 min instead of 90 min. With this approach, SSO_2_ therapy reached better long-term clinical outcomes—composite of all cause-death or new-onset HF or HF hospitalization at 1 year—compared with the control group (0% vs. 12.3%; *p* = 0.01). Also, the rate of stent thrombosis was numerically higher in the control group compared with the SSO_2_ group (1.2% vs. 4.9%, *p* = 0.17) [64].

Based on this study, the US FDA approved in 2019 the SSO_2_ therapy for patients with anterior STEMI presenting within 6 h of symptoms. However, appropriately powered randomized trials are needed to prove the effect of SSO_2_ treatment in patients with anterior STEMI. Meanwhile, more research is under way to expand SSO_2_ indications in STEMI patients. The Incorporating Supersaturated Oxygen in Shock (ISO-SHOCK trial: NCT04876040) study is a multicenter randomized that aims to evaluate the safety and feasibility of SSO_2_ therapy in 60 patients presenting with STEMI and cardiogenic shock.

## 7. Therapeutic Hypothermia

Myocardial cooling with mild therapeutic hypothermia (32–35 °C) has been effective in experimental models as a cardioprotective strategy. Hypothermia decreases myocardial metabolic demands and attenuates all the inflammatory response involved in reperfusion injury [65]. Large animal studies have shown that hypothermia during myocardial infarction reduces infarct size when applied as early as possible during ischemic period—and not after the onset of reperfusion [66,67]. The inability to achieve myocardial cooling before reperfusion has been one of this strategy’s major concerns.

In the RAPID MI-ICE trial (18 patients), hypothermia was successfully achieved—using intravascular cooling combined with cold saline—in 100% of patients with minor reperfusion delay. Despite initial encouraging results (37% reduction in infarct size; *p* = 0.04) [68]; this method failed to demonstrate a significant reduction in infarct size in the larger CHILL-MI trial (120 patients). However, an exploratory analysis of early presenters with anterior STEMI indicated some benefit in terms of infarct reduction and heart failure incidence [69]. Thus, the recent COOL AMI EU Pivotal Trial was carried out to prove differences in anterior STEMI patients with <4.5 h of symptoms. In this study, the use of 1 L intravenous infusion of cold saline and intravascular ZOLL TM Proteus Cooling System did not reduce percentage of infarct size (21.3% vs. 20.0%; *p* = 0.540). Also, although not statistically significant (8.6% vs. 1.9%; *p* = 0.12), there was an excess of major adverse events—including atrial fibrillation, ventricular tachycardia/fibrillation, and stent thrombosis. Actually, the study was stopped prematurely due to a 44-min increase in total ischemic delay in the hypothermia group [70].

These systemic cooling techniques face many logistical challenges that limit the potential benefit of hypothermia in acute myocardial infarction—not reaching target temperature fast enough. In order to overcome this limitation, the use of selective intracoronary hypothermia has been developed [71]. A pressure/temperature wire is introduced in the coronary artery and an over-the-wire balloon is subsequently inflated to keep the artery occluded. Firstly, saline at room temperature is infused for 10 min into the infarct area at a flow rate of 15–30 mL/min (occlusion phase). Secondly, the balloon is deflated allowing reperfusion; but infusion with saline at 4 °C is maintained for 10 more minutes (reperfusion phase). Distal coronary temperature is continuously monitored by a pressure/temperature guidewire to maintain a distal coronary temperature of between 4 °C and 6 °C below body temperature.

After having proved safety and feasibility a pilot trial, the EURO-ICE trial (NCT03447834) has been designed to study differences in infarct size in 200 patients comparing selective intracoronary hypothermia compared with standard PCI [72].

## 8. Pressure-Controlled Intermittent Coronary Sinus Occlusion

The origins of transcoronary sinus interventions can be found in the first half of the 20th century in the Beck’s procedure that arterialized the coronary venous vasculature [73]. Later, the percutaneous translation of the concept “perfusing the myocardium by the back door” via the coronary sinus was developed in the early 1980s [74].

**Figure 2:**
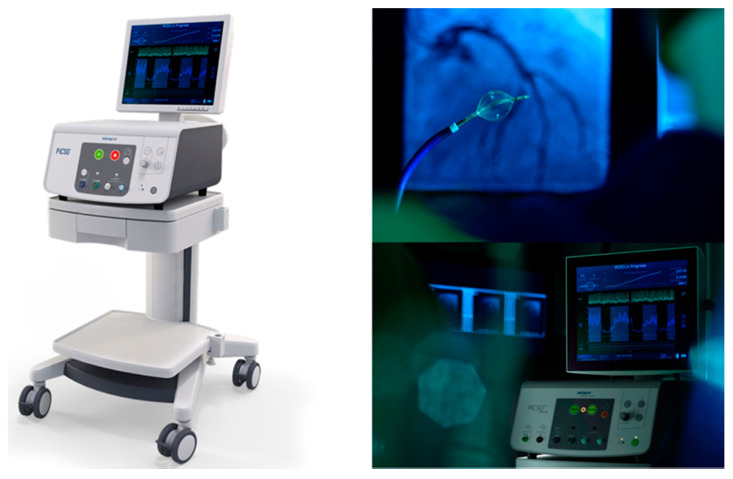
Pressure-controlled intermittent coronary sinus occlusion (PICSO) impulse system. A balloon-tipped catheter is introduced in the coronary sinus. The catheter, which is connected to a console, is able to monitor coronary sinus pressures and automatically inflates and deflates in a periodic way. Images courtesy of Miracor Medical Systems GmbH, Vienna, Austria.

Pressure-controlled intermittent coronary sinus occlusion (PICSO) is a technique that periodically occludes the coronary sinus with a balloon-tipped catheter that is introduced via the femoral vein. This catheter has a sensor—placed at the coronary sinus ostium—to monitor coronary sinus pressure. Automatically, during coronary occlusion of the coronary sinus, there is a progressive increase in systolic venous pressure until a “plateau pressure” is reached—resulting in blood accumulation in the venous system and an increase of diastolic coronary sinus pressure. Then, the balloon is deflated and the venous pressure decreases suddenly (creating a gradient) that allows venous drainage (Figure 2) [75].

Therefore, PICSO therapy has been proposed as an additional tool for acute myocardial infarction treatment. PICSO is intended to reduce both ischemia and reperfusion injury—and ultimately infarct size—via different mechanisms:

During occlusion period (5–15 s) venous flow is redistributed from normally perfused areas towards the ischemic zones through heterocoronary and homocoronary collaterals activation. Also, there is a ‘plasma skimming phenomenon’ in the venous microcirculation of the occluded territory—the systolic pressure increase pushes the blood plasma from the larger veins to venules—leading to a better perfusion in this area with plasma rich in oxygen and metabolites.

In the balloon release period (3–4 s) the sudden drop in venous pressure creates a gradient that allows wash out of thrombotic debris, toxic metabolites, and myocardial edema from the microcirculation [76].

Additionally, it is hypothesized that the cyclical change in coronary sinus pressure may activate endogenous pathways of cardiac repair. These temporary pressures elevations are believed to induce mechanotransduction and activation of vascular cells with regional release of growth factors, vasodilator substances, and miRNAs into microcirculation [77]. Furthermore, in patients with chronic heart failure who underwent successful cardiac resynchronization therapy; PICSO has proved a significant induction of cell proliferation and expression of several relevant miRNAs into cardiac veins. These results support the “cardiac regeneration induced by mechanotransduction” theory with PICSO treatment [78].

The effect of PICSO on infarct size has been consistently positive in various animal models with a significant reduction in infarct size related to coronary sinus occlusion compared with the control group—as a meta-analysis of seven studies revealed (mean infarct size 48.7% and 78.8%; mean difference of 29%; *p* < 0.001) [79]. The Prepare PICSO study was the first-in-man study where PICSO was evaluated. In this non-randomized single-center study 15 patients with stable angina pectoris underwent PICSO therapy in the midst of a left anterior descending artery PCI. PICSO treatment was delivered without any device-related adverse events and resulted in an increase in LAD wedge pressure [80]. Later, the same group enrolled 30 anterior STEMI patients in whom PICSO therapy was applied during 90 min. PICSO was safe in the STEMI context and exploratory analyses suggested favorable trends in MVO and infarct size reduction in a dose-dependent manner—pointing out the importance of an adequate “PICSO quantity”. However, there was not a significant reduction on infarct size in PICSO group. Notably, some limitations were found in this study: PICSO treatment was not performed in 1/3 of cases, 40% of patients presented with TIMI flow >1 and the PICSO system was implanted after stenting in all patients [81]. The OxAMI-PICSO study focused on anterior STEMI with higher microvascular dysfunction—using an index of microvasculature resistance (IMR) > 40. In these selected high-risk STEMI patients (*n* = 25), PICSO improved microvascular function 24–48 h post-PCI and reduced infarct size at six months compared with the control group (26% vs. 33%; *p* = 0.006) [82]. In the also non-randomized PICSO-ACS study 45 STEMI patients were treated with PICSO and compared with an historical cohort with a propensity score-matched analysis. In this case, there was a 33% relative reduction in infarct size at 5 days (21% vs. 14%; *p* = 0.023) [83].

These promising studies have resulted in the setup of the first randomized clinical studies with PICSO therapy. Currently, the multicenter randomized PICSO AMI I trial (NCT03625869) is recruiting anterior STEMI patients (*n* = 144) with TIMI 0–1 to assess reduction in infarct size at 5 days.

In the current treatment of STEMI, there is no discussion about the “time is muscle” paradigm. Thus, in the most recent studies, PICSO therapy is implemented after restoration of coronary blood flow but before stenting—preparing the injured myocardium for the following damage caused by stent implantation without delay in door-to-balloon time. However, timing of PICSO treatment—and other cardioprotective therapies—remains unclear; specially if we attend to reperfusion injury pathophysiology, which is rapidly harmful in the first minutes after flow restoration [15].

## 9. Conclusions

Although primary PCI has greatly improved outcomes in patients with myocardial infarction and it is considered essential in STEMI treatment, a remarkable group of patients still suffer significant myocardial damage with substantial mortality and morbidity in the context of the later development of heart failure due to ventricular dysfunction. This poorer prognosis is mainly related to the extent of infarct size. Many factors contribute directly to infarct size magnitude: not only a promptly PCI to open the artery and achieve myocardium reperfusion is important, but also some of the pathophysiological phenomena implied in the myocardial infarction process—such as distal embolization, reperfusion injury and microvascular dysfunction—can be very harmful and cause myocardial injury.

As a result, given a better knowledge of all the mechanisms involved in the infarct size determinants; novel therapeutic strategies have been identified for potentially increase myocardial salvage and thus improve clinical results. In this review, we focus on different mechanical therapeutic techniques beyond stenting in the interventional area. Encouraging data in animal models generated high expectations; however, transferring these findings to the clinical scene has been challenging with mixed results for infarct size reduction or improved clinical endpoints (summarized in Table 1). Thus, to get clearer conclusions in the clinical field it is essential to improve the design of clinical cardioprotection studies—including patient inclusion, time and dose of the intervention, or use of relevant and standardized endpoints. While some of these practices have been abandoned for the routine clinical practice, such as thrombectomy, distal protection devices, or IABC; other novel therapies are being tested in randomized clinical trials with greater sample sizes and better designs (summarized in Table 2). All of this new evidence will offer more clarity in the unmet therapeutic need of STEMI optimized treatment.

## Figures and Tables

**Table 1:** Summary of key studies of novel interventional treatments in STEMI patients.

Treatment	Study and Design	*n*	Intervention	Main Results	Notes	Ref.
Thrombus aspiration	TASTE (2014) RCT	7244	Thrombus aspiration	No difference in 1-year mortality		[25]
	TOTAL (2015) RCT	10732	Thrombus aspiration	No difference in CV death, recurrent MI, cardiogenic shock, NYHA class IV HF in 180 days	↑ stroke in 30 days (0.7% vs. 0.3%) *	[26]
Sono thrombolysis	Mathias et al. (2019) RCT	100	Diagnostic ultrasound with contrast agent plus HMI pulses	58% ↑ angiographic recanalization * 27% ↓ MI size by CMR at 72 h * 11% ↑ LV ejection fraction at 6 months *		[32]
Distal protectiondevices	EMERALD (2005) RCT	501	Aspiration distal microcirculatory protection system	No difference in ST resolution, MI size at 5 days or MACE at 6 months		[35]
Ischemic post conditioning	Khalili et al. (2014) Metanalysis	1545	Coronary inflation/deflation cycles with angioplasty balloon	No difference in ST resolution, MI size, mortality, recurrent MI, stent thrombosis or MACE	15 RCTs reviewing clinical outcomes	[45]
	DANAMI 3-iPOST (2017) RCT	1234	4 repeated 30-s balloon occlusions followed by 30-s reperfusion	No difference in all-cause mortality and HF hospitalization at 38 months	44% ↓ all-cause mortality and HF hospitalization in patients without thrombectomy *	[48]
Ventricular unloading	CRISP-AMI (2011) RCT	337	IABC unloading before PCI	No difference in MI size by CMR at 3–5 days		[53]
	DTU-STEMI pilot trial (2019) RCT	50	Impella CP^®^ unloading during 30 min before primary PCI	No difference in MACE or MI size by CMR at 30 days	Safety and feasibility trial	[58]
SSO_2_	AMIHOT-II (2009) RCT	301	Intracoronary SSO_2_ in LAD during 90 min	26% ↓ MI size by Tc-99m-sestamibi SPECT and non-inferior MACE at 30 days (3.8% vs. 5.4%) *	↑ Hemorrhagic complications and stentthrombosis	[63]
	IC-HOT (2021) RCT	100	“Optimized” intracoronary SSO_2_ therapy in LAD during 60 min	↓ all-cause 1 year mortality or new HF onset/hospitalization (0.0% vs. 12.3%) *	No difference in stent thrombosis between groups	[64]
Coronary hypothermia	COOL-AMI EU (2021) RCT	111	Hypothermia with intravascular cooling system	No difference in MI size by CMR ↑ MACE in the hypothermia group	Discontinuation due to 44-min ↑ ischemic time in hypothermia group	[70]
PICSO	Ox-AMIPICSO (2018) Observational	105	PICSO after flow restoration andbefore stenting during 33 min	↑ microvascular function and 21% ↓ MI size by CMR at 6 months *	Patients were stratified based on IMR	[82]
	PICSO in ACS (2020)Observational	92	PICSO after flow restoration andbefore stenting during 30 min	33% ↓ MI size by CMR at 5 days *		[83]

CMR: Cardiac magnetic resonance; CV: Cardiovascular; HF: Heart failure; HMI: High mechanical index; IABC: Intra-aortic balloon counterpulsation; IMR: Index of microcirculatory resistance; LAD: Left anterior descending; LV: Left Ventricle; MACE: Major adverse cardiac events; MI: myocardial infarction; PICSO: Pressure-controlled intermittent coronary sinus occlusion; PCI: Percutaneous coronary intervention; RCT: Randomized clinical trial; SPECT: Single-photon emission computed tomography; SSO_2_: Supersaturated oxygen; STEMI: ST-segment-elevation myocardial infarction. * *p* < 0.05.

**Table 2:** Major ongoing clinical trials investigating novel therapeutic strategies in STEMI patients.

Treatment	Study	Estimated Enrollment (*n*)	Condition	Intervention	Primary Endpoint	Estimated Completion Date
Sono thrombolysis	SONOSTEMILYSIS trial	60	High-risk STEMI (>2 mm in ECG) undergoing fibrinolysis	Diagnostic ultrasound with contrast agent plus HMI pulses vs. diagnostic ultrasound plus standard therapy alone	Complete ST- segment resolution 90 min post- fibrynolisis	May 2023
Ischemic post conditioning	iPOST2 trial	1800	STEMI with TIMI flow 0–1	Ischemic postconditioning with balloon (4 cycles 60 s reperfusion/60 s re-occlusion) without thrombectomy vs. standard PCI	All-cause mortality or HFhospitalization	January 2024
Left ventricle unloading	STEMI DTU pivotal trial	668	Anterior STEMI	Impella CP^®^ placement through a femoral arterial sheath and activation during 30 min prior to primary PCI vs. standard PCI	Infarct size 3–5 days post-procedure by CMR	October 2027
SSO_2_	ISO SHOCK trial	60	STEMI withcardiogenic shock	PCI + Impella CP^®^ + 60-min adjunctive reperfusion of SSO_2_ into culprit artery vs. PCI + Impella CP^®^	All-cause mortality at 30 days	June 2025
Coronary hypothermia	EURO ICE trial	200	Anterior STEMI with TIMI flow 0–1	Selective intracoronary hypothermia during 20 min (10 min of occlusion phase and 10 min of reperfusion phase) followed by PCI vs. standard PCI	Infarct size 3 months after STEMI by CMR	January 2022
PICSO	PICSO AMI I trial	144	Anterior STEMI with TIMI flow 0–1	Coronary sinus cannulation through femoral vein and PICSO placement, followed by stenting; then PICSO therapy during 45 min vs. standard PCI	Infarct size 5 days after STEMI by CMR	July 2025

CMR: Cardiac magnetic resonance; HF: Heart failure; HMI: High mechanical index; PICSO: Pressure-controlled intermittent coronary sinus occlusion; PCI: Percutaneous coronary intervention; SSO_2_: Supersaturated oxygen; STEMI: ST-segment-elevation myocardial infarction; TIMI: Thrombolysis in Myocardial Infarction.

## Data Availability

Not applicable.
